# Understanding the Role of Temperature and Drain Current Stress in InSnZnO TFTs with Various Active Layer Thicknesses

**DOI:** 10.3390/nano10040617

**Published:** 2020-03-27

**Authors:** Dapeng Wang, Mamoru Furuta, Shigekazu Tomai, Koki Yano

**Affiliations:** 1 Key Laboratory of Applied Surface and Colloid Chemistry, Ministry of Education; Shaanxi Key Laboratory for Advanced Energy Devices; Shaanxi Engineering Lab for Advanced Energy Technology, School of Materials Science and Engineering, Shaanxi Normal University, Xi’an 710119, China; 2 School of Environmental Science and Engineering, Kochi University of Technology, Kami, Kochi 782-8502, Japan; 3 Center for Nanotechnology in Research Institute, Kochi University of Technology, Kami, Kochi 782-8502, Japan; 4 Advanced Technology Research Laboratories, Idemitsu Kosan Co. Ltd., Sodegaura, Chiba 299-0293, Japan; shigekazu.tomai.6560@idss.co.jp (S.T.); koki.yano@idemitsu.com (K.Y.)

**Keywords:** drain current bias, test temperature, InSnZnO, TFT device, channel layer thickness

## Abstract

Thin-film transistor (TFT) devices composed of metal oxide semiconductors have attracted tremendous research attention globally in recent years. Owing to their ability to offer mobility, metal oxide semiconductor materials can enable high-performance TFTs for next-generation integrated display devices. Nevertheless, further breakthroughs of metal oxide TFTs are mainly obstructed by their long-term variability, the reason for which is not yet fully understood. Herein, TFTs based on InSnZnO (ITZO) with various thicknesses (*T*_ITZO_) were prepared and their long-term stabilities under test temperatures and drain current stress were investigated. The results indicate that ITZO TFTs exhibit outstanding electrical properties regardless of the *T*_ITZO_, including a high saturated mobility of over 35 cm^2^V^−1^s^−1^ and sharp subthreshold swing. Note that the transfer and output characteristic curves of the device with a thick *T*_ITZO_ of 100 nm express an abnormal current surge when high gate and drain voltages are exerted, which is attributed to the floating body effect, caused when the imposed electric field induces impact ionization near the drain side. More interestingly, these drain current stress results further suggest that the abnormal shift behavior of the electrical properties of the ITZO TFTs with a *T*_ITZO_ of greater than 75 nm is observed to deteriorate gradually with increasing temperature and drain current bias. This study addresses that such a degradation effect should be restrained for the operation of high-mobility devices.

## 1. Introduction

Metal oxide semiconductors have recently emerged as extremely sought-after materials for thin-film transistor (TFT) applications [1,2,3,4,5,6]. Over the past decade, the mobility (*μ*) of TFTs employing these semiconductors, such as InGaZnO, has greatly increased, and is comparable to that of polysilicon-based devices [7]. These metal oxide semiconductors also possess promise for various optoelectronic applications, including photodetectors and solar cells [8,9,10,11]. Inspired by the achievement of metal oxide films, another category of these materials, called InSnZnO (ITZO), was studied by our group in 2012 [12]. From the perspective of potential applications in flat panel displays (FPDs), the ITZO TFTs have been gifted with desirable electrical properties, especially the high *μ* of >30 cm^2^V^−1^s^−1^, which originates from the 5 *s* orbital overlaps of In and Sn atoms [13].

Besides the tremendous developments of metal oxide semiconductors and TFT structures, an intensive understanding of the charge-carrier transport processes is beneficial to further improving the TFT characteristics. In addition, the systematic carrier motion model extending to the internal active layer, which is the core of operating TFT devices, still needs to be studied. Adjusting the metal oxide thickness is an intuitive and effective parameter of the model to investigate the charge transport processes in the bulk of the active layer with [14,15,16], while leaving the condition of the adjacent interfaces almost unchanged. Apart from the design and fabrication of TFT architectures, the measurement approach is also a crucial step to set up the fundamental physical framework [17]. In addition, in terms of TFT devices, the long-term stabilities are critical to drive TFT-contained integrated circuits in FPDs [18,19]. Moreover, the drain current stress is a common working procedure in current-biased TFTs [20]. 

In this regard, here the typical TFT device structure is considered to verify the universality of the overall framework. On the basis of the device’s architecture, channel layers with different thicknesses (*T*_ITZO_) are then designed. To evaluate the device’s stability, the measurement conditions and working environment are taken into consideration. More importantly, to understand the carrier transport mechanism in the devices, both the channel layer thickness and drain current stress (DCS) should be fully considered.

## 2. Experimental

**Figure 1:**
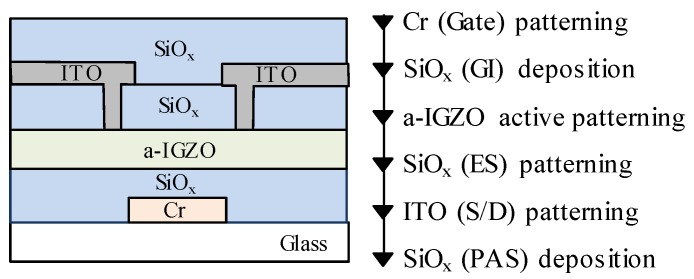
Device architecture and preparation process of the ITZO TFT.

Figure 1 displays the device architecture and fabrication procedure of ITZO-based TFT, which refers to our previous publication [21]. The chromium gate electrode was prepared on the glass substrate, and the SiO*_x_* gate insulator with a thickness of 150 nm was formed by plasma-enhanced chemical vapor deposition (PECVD). For the active layers, ITZO films with the thicknesses of 45, 75, and 100 nm were prepared by DC magnetron sputtering with a mixed gas of Ar/O_2_ = 15/15 sccm at a deposition pressure of 1 Pa. Then, a 200 nm thick SiO*_x_* etch-stopper was deposited by PECVD, and indium-tin-oxide source and drain electrodes were formed. Finally, a SiO_x_ passivation layer was fabricated by PECVD. After the fabrication of devices with channel widths of 50 μm and lengths of 20 μm, the TFTs were post-treated in a nitrogen atmosphere at 350 °C for 1 h. All the current–voltage (I–V) results were evaluated by an Agilent 4156C precision semiconductor parameter analyzer in air.

## 3. Results and Discussion

**Figure 2:**
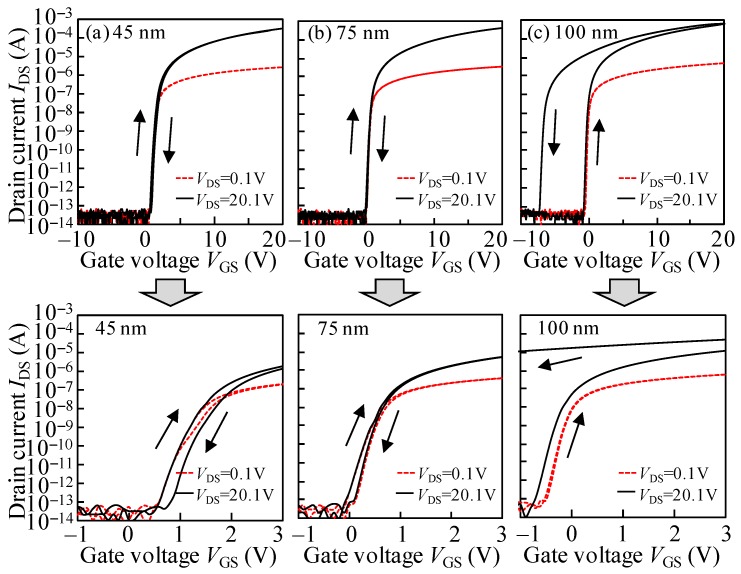
Transfer characteristics of TFT devices with different *T*_ITZO_ of (**a**) 45, (**b**) 75, and (**c**) 100 nm evaluated at a *V*_DS_ of 0.1 and 20.1 V.

The transfer curves of TFT devices with different *T*_ITZO_ were evaluated at a drain voltage (*V*_DS_) of 0.1 and 20.1 V, as shown in Figure 2. The linear (*μ*_lin_) and saturated (*μ*_sat_) mobilities were estimated on the basis of previous literature [21]. The turn-on voltage (*V*_ON_) was extracted from the gate voltage (*V*_GS_) at a drain current (*I*_DS_) of 1 nA. The hysteresis (Δ*V*_H_) was calculated from the *V*_ON_ difference value between the forward and reverse scans of the transfer curves. The subthreshold swing (SS) was defined to be d*V*_GS_/dlog_10_(*I*_DS_). The respective parameters were extracted from the forward sweep, as tabulated in Table 1. 

For the device with a 45 nm *T*_ITZO_, a *μ*_lin_ of 28.76 cm^2^V^−1^s^−1^, *μ*_sat_ of 35.23 cm^2^V^−1^s^−1^, *V*_ON_ of 1.19 V, Δ*V*_H_ of 0.22 V, and SS of 169 mV/dec. were calculated. The observed results indicate that ITZO-based TFTs exhibit outstanding electrical characteristics which are generally superior to the results of IGZO-based TFT devices [22]. The *μ*_lin_ gradually rose to 33.27 and 46.36 cm^2^∙V^−1^∙s^−1^ when the *T*_ITZO_ thickened to 75 and 100 nm, respectively. For the mobility extracted from the saturation region, the *μ*_sat_ significantly increased to 46.90 cm^2^∙V^−1^∙s^−1^ for the 75 nm *T*_ITZO_ device, and sharply soared to 130.10 cm^2^∙V^−1^∙s^−1^ as the *T*_ITZO_ further thickened to 100 nm. It was found that the *I*_DS_ values significantly increased with the increase in *T*_ITZO_. In general, the increase in the *μ* and *I*_DS_ of the thicker channel TFT was attributed to the increase in carrier concentration and decrease in the density of subgap states, which were based on the percolation conduction and multiple trapping and release model [23,24]. Combined with the transfer curves observed from the 100 nm *T*_ITZO_ TFT measured with a *V*_DS_ of 20.1 V and *V*_GS_ of ~20 V, we can conclude that the calculated *μ*_sat_ hardly represents the intrinsic properties of the ITZO material. In the sections of DCS-induced instabilities, for the devices with thicker *T*_ITZO_ TFT and measurement modes with a high *V*_DS_ and *V*_GS_ bias, this conclusion of the sudden increase in the *μ*_sat_ was further confirmed. In addition, the *V*_ON_ slightly shifted to 0.43 and −0.33 V as the *T*_ITZO_ gradually thickened to 75 and 100 nm, respectively. Because the free carrier content in the bulk is directly proportional to the thickness of samples [25], the *V*_ON_ changed in the negative *V*_GS_ direction with the increase in the *T*_ITZO_. The negligible clockwise hysteresis was observed for the devices with a *T*_ITZO_ of 45 and 75 nm. As the *T*_ITZO_ was enlarged to 100 nm, an abnormal anticlockwise hysteresis of −6.38 V was obtained. In other words, the *V*_GS_ was swept forward from −10 to 20 V with 20.1 V *V*_DS_. 

On the basis of the previous publication [26], high-μ TFTs experiencing drain current stress may induce the floating body effect that impacts ionization, producing electron-hole pairs in the drain side. The generated holes drift to the ITZO/etch-stopper interface due to the longitudinal electric field and flow to the source region due to the transverse electric field. Furthermore, for the TFTs with a thick *T*_ITZO_, superfluous holes are trapped in the source area of the back-channel interface, naturally causing the transfer curve’s negative shift. Therefore, in terms of 100-nm-thick ITZO TFTs, a tremendous shift in the negative *V*_GS_ direction was observed in the transfer properties, leading to the great hysteresis. Interestingly, the SS values manifest the gradient descent as 130 and 88 mV/dec. as the *T*_ITZO_ increases to 75 and 100 nm, respectively. This behavior is due to the fact that the densities of interface traps at the ITZO/etch-stopper area are lessened as the *T*_ITZO_ increases [27]. It is commonly known that the SS is the direct parameter by which to evaluate the integration of trap state densities in the ITZO film and corresponding interfaces [28]. The results further imply that the interface quality is directly related to the device properties.

**Figure 3:**
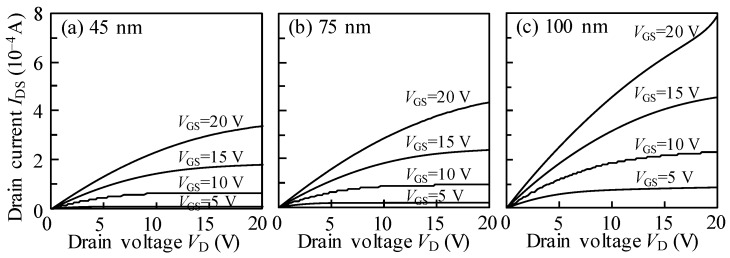
Output characteristics of TFT devices with various *T*_ITZO_ of (**a**) 45, (**b**) 75, and (**c**) 100 nm.

Figure 3 plots the typical output curves of ITZO TFTs with various *T*_ITZO_. The *V*_GS_ was altered from 5 to 20 V in increments of 5 V. All devices exhibited standard n-type field-effect transistor behavior. When the TFTs were switched on, the output properties presented enhancement-mode operation with a clear pinch-off phenomenon regardless of the *T*_ITZO_. For the devices based on 45 and 75 nm *T*_ITZO_, the current saturation response was evidently observed. The saturation current under the identical *V*_GS_ and *V*_DS_ biases gradually increased with the *T*_ITZO_. For 100 nm *T*_ITZO_ TFT, a similar current saturation response was also obtained when the *V*_GS_ was controlled below 15 V. However, the output characteristics expressed a current increase under high drain and source voltages of 20 V. This phenomenon can be explained by the kink effect that impacts ionization-induced hole flow towards the source side. Moreover, electrons were injected into the channel from the source side and collected at the drain region. The extra drain current accelerated the impact ionization probability. Consequently, this effect led to a sharp rise in the output characteristics, which was in agreement with the results for the transfer characteristics. 

**Figure 4:**
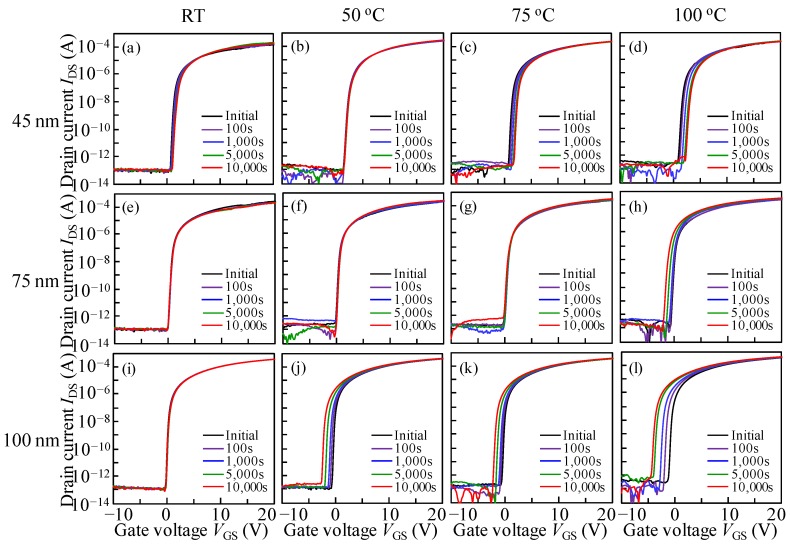
Evolution of transfer characteristics as a function of DCS (*V*_GS_ = *V*_DS_ = 10 V) duration for 10^4^ s for the devices with a *T*_ITZO_ of 45 nm measured at test temperatures of (**a**) 25, (**b**) 50, (**c**) 75, and (**d**) 100 °C; with a *T*_ITZO_ of 75 nm measured at test temperatures of (**e**) 25, (**f**) 50, (**g**) 75, and (**h**) 100 °C; and with a *T*_ITZO_ of 100 nm measured at test temperatures of (i) 25, (**j**) 50, (**k**) 75, and (**l**) 100 °C.

**Figure 5:**
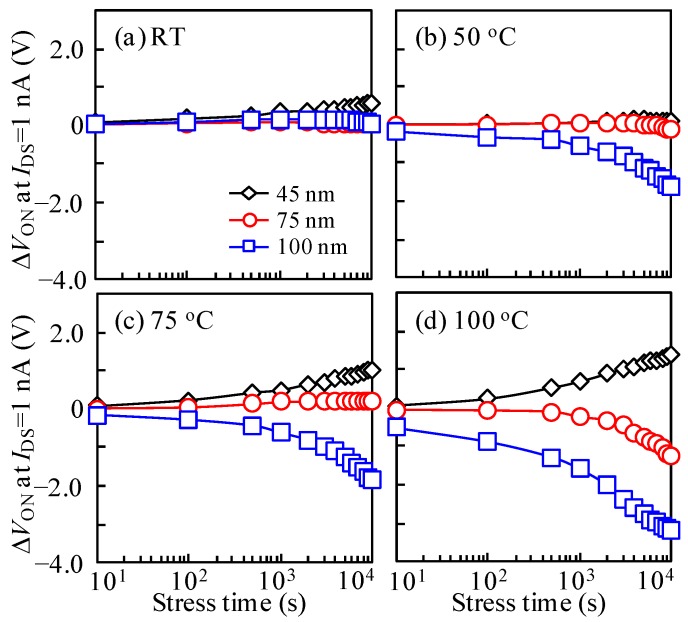
Variation in the Δ*V*_ON_ with DCS duration for the TFT devices measured at test temperatures of (**a**) RT, (**b**) 50, (**c**) 75, and (**d**) 100 °C.

To comprehend the influence of measurement temperatures on the DCS-induced instability of ITZO TFTs with various *T*_ITZO_, the test platform temperatures were set to 25, 50, 75, and 100 °C, and the DCS of *V*_GS_ = *V*_DS_ = 10 V was fixed for 10^4^ s. To avoid the abnormal transfer curve shift occurring, a *V*_DS_ of 10.1 V was chosen. The variations in the transfer properties of ITZO TFTs with different *T*_ITZO_ were studied under DCS at various test temperatures (Figure 4). Correspondingly, the changes in *V*_ON_ (Δ*V*_ON_) accompanied by DCS time under various test temperatures are shown in Figure 5. It is noted that the current fluctuations in the OFF-current region are observed in some I-V curves. This phenomenon may be due to the poor shielding effect of the test platform during the measurement process. Even so, a current level of below 10^−13^ A had no effect on the DCS evaluation. For the devices measured at room temperature, all of the transfer curves exhibited stable behaviors even throughout a long-term duration, suggesting that a high quality GI layer has been fabricated, and the electrons were seldom captured at the ITZO/GI interface. As the test temperature was raised to 50 °C, the electrical properties of 45 and 75 nm *T*_ITZO_ TFTs still presented a steady state. However, in terms of the device with a thick *T*_ITZO_ of 100 nm, a parallel shift in the transfer characteristics with a Δ*V*_ON_ of −1.60 V was observed after the DCS duration. The results indicate that a high temperature promotes thermal movement of the electron, contributing to the occurrence probability of impact ionization. As the temperature was further adjusted to 75 °C, the transfer curves of the TFT with 45 nm *T*_ITZO_ shifted by 1.02 V towards a positive *V*_GS_ direction with unchanged SS during the 10^4^ s stress time, demonstrating that the generated electrons induced by thermal activation were trapped at the front interface. It was noted that the device with a 75 nm *T*_ITZO_ still maintained excellent electrical stability. In the case of the 100 nm *T*_ITZO_ device, the motion behavior was enlarged and the Δ*V*_ON_ was aggravated to −1.85 V. When the platform temperature was increased to a harsh condition of 100 °C, the transfer curves of the TFT with a 45 nm *T*_ITZO_ continually changed by 1.40 V after the DCS duration. In contrast, for the *T*_ITZO_ thicknesses of 75 and 100 nm, the transfer characteristics showed parallel movement in a negative way and the Δ*V*_ON_ values were respectively exacerbated to −1.27 and −3.18 V over the whole DCS time. These results state that temperature plays a key role in exciting electron movement, thereby concretizing and enlarging electron-related effects.

**Figure 6:**
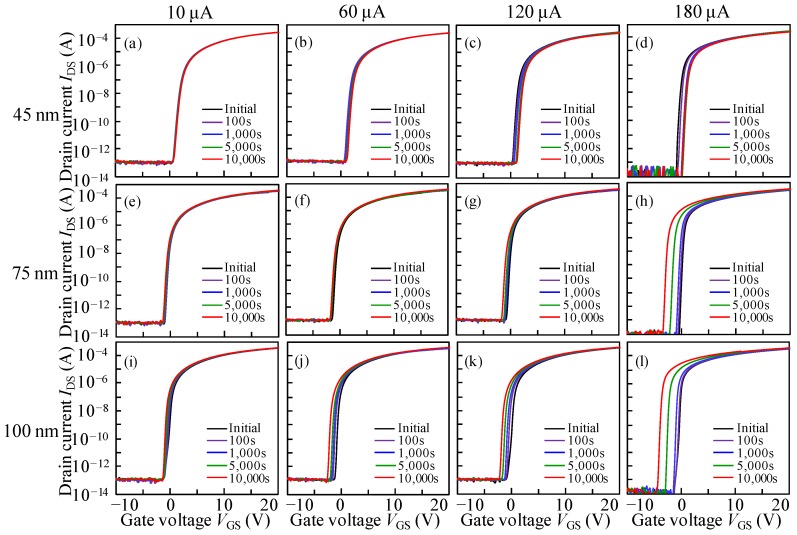
Evolution of transfer characteristics as a function of 10^4^ s DCS duration with the test temperature of 50 °C for the devices with a *T*_ITZO_ of 45 nm under *I*_DS_ stresses of (**a**) 10, (**b**) 60, (**c**) 120, and (**d**) 180 μA; with a *T*_ITZO_ of 75 nm under *I*_DS_ stresses of (**e**) 10, (**f**) 60, (**g**) 120, and (**h**) 180 μA; and with a *T*_ITZO_ of 100 nm under *I*_DS_ stresses of (**i**) 10, (**j**) 60, (**k**) 120, and (**l**) 180 μA.

**Figure 7:**
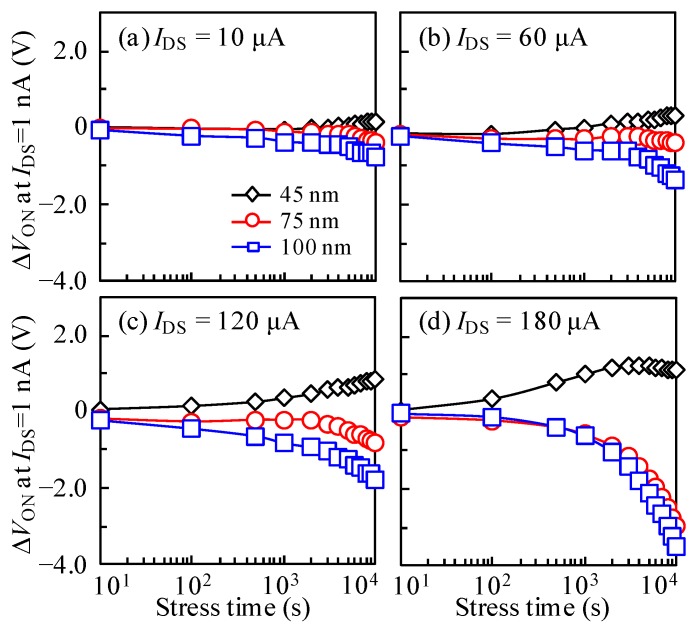
Variation in the Δ*V*_ON_ with DCS duration for the TFT devices measured at *I*_DS_ stresses of (**a**) 10, (**b**) 60, (**c**) 120, and (**d**) 180 μA.

To further study DCS-caused instability of the ITZO TFTs with various *T*_ITZO_, the *I*_DS_ values of 10, 60, 120, and 180 μA adjusted by the gate and drain voltages (*V*_GS_ = *V*_DS_) were applied at 50 °C up to the stress duration of 10^4^ s. It should be noted that these *I*_DS_ values are much higher than those applied in conventional active-matrix organic light-emitting diodes displays (~2 μA) [29]. Figure 6 displays the corresponding variation in the transfer curves of the devices. Note that the transfer characteristics of all TFTs under various *I*_DS_ stresses exhibit a parallel shift without SS degradation. The corresponding Δ*V*_ON_ fluctuations are plotted in Figure 7. All devices exhibit acceptable reliability under 10 μA DCS irrespective of the *T*_ITZO_, implying that few electron trapping sites exist in the ITZO bulk and at the ITZO/GI interface. Interestingly, with the increase in the *I*_DS_ stress, a Δ*V*_ON_ with a significant *T*_ITZO_ dependence was observed. For the 45 nm *T*_ITZO_ TFT, the *V*_ON_ positively shifted with the increase in the *I*_DS_ stress. The positive shift behavior was enlarged by increasing the stress duration. For the 180 μA *I*_DS_ stress, the transfer curves parallel shifted by 1.21 V towards the positive *V*_GS_ direction under the stress duration of 3000 s. In the subsequent stress time, the Δ*V*_ON_ shift was saturated in a short stress time, and then slightly moved back to 1.10 V. In contrast, when the thickness increased to 75 and 100 nm, the transfer curves completely changed in the negative *V*_GS_ direction irrespective of the *I*_DS_ stress. Under the 180 μA *I*_DS_ stress, the Δ*V*_ON_ of the TFTs with *T*_ITZO_ of 75 and 100 nm decreased by −2.98 and −3.50 V, respectively. The results demonstrate that, under identical *I*_DS_ stress, the negative *V*_ON_ shift is accelerated with the increase in *T*_ITZO_. 

**Figure 8:**
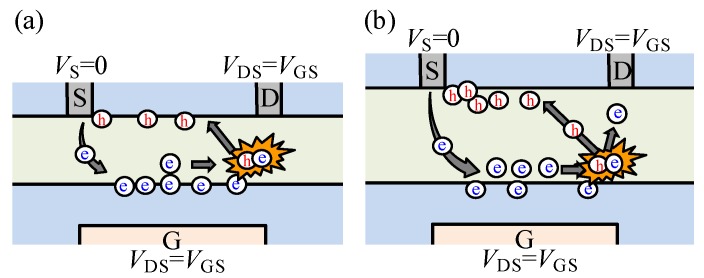
The schematic diagram of impact ionization mechanism of DCS-originated instability in the devices with a *T*_ITZO_ of (**a**) 45 and (**b**) 75 and 100 nm.

Generally, the positive *V*_ON_ shift was due to electron trapping at the front-channel interface, while the *V*_ON_ shift to the negative *V*_GS_ direction was due to hole trapping at the active layer/etch-stopper interface under the *I*_DS_ stress. For the 45 nm *T*_ITZO_ device under low *I*_DS_ (< 180 μA) stress (*V*_DS_ = *V*_GS_), electrons drifted to the gate insulator/ITZO region. Simultaneously, electron trapping occurred immediately at the gate insulator/ITZO interface, as illustrated in the corresponding schematic diagram (Figure 8a).

As the *I*_DS_ stress increases, the electron trapping probability is enhanced. When the *I*_DS_ increases to 180 μA, electron trapping is the main origin of the positive *V*_ON_ shift within the stress duration of 1000 s. Consequently, a saturated trapping behavior is then observed. When the stress time exceeds 3000 s, hole trapping seems to become a major reason for the slightly negative *V*_ON_ shift. It can be explained by the impact ionization phenomenon [21], which induces the generation of an electron-hole pair. Consequently, the excited charges are trapped at the front- and back-channel interfaces. However, the electron trapping seems to become saturated at the gate insulator/ITZO region. Therefore, the extra electrons are drained from the drain electrode. The holes generated near the drain side migrate to the source region along the back-channel interface and accumulate near the source region. As a result, the *V*_ON_ shifts from a positive to negative *V*_GS_ direction. When the *T*_ITZO_ increases to 75 nm and more, a clear negative *V*_ON_ shift is observed under various *I*_DS_ stresses, demonstrating that hole trapping at the ITZO/etch-stopper interface is the main cause of the negative *V*_ON_ shift. With the increase in the *T*_ITZO_, the negative *V*_ON_ shift is accelerated under the identical *I*_DS_ stress. The results demonstrate that, for the thicker *T*_ITZO_ TFTs, electrons injected from the source will experience a longer duration prior to reaching the gate insulator/ITZO interface under the vertical electric field. Simultaneously, electrons are subjected to a driving force from the lateral electric field. It is noted that the identical *I*_DS_ bias is controlled with different *V*_GS_ = *V*_DS_ values for various *T*_ITZO_ TFTs. Therefore, the probability of electron-induced impact ionization is greatly increased with sufficient space and time, contributing to more excited carriers (Figure 8b). It is noted that the forward and reverse transfer curves were compared after DCS, and the similar transfer curves were obtained without SS degradation. Therefore, the results suggest that the observed phenomenon is hard to attribute to the increase in the donor-like states near the source side of the TFTs.

On the basis of the above-mentioned discussions, the obtained results suggest that, in the high-μ devices, the DCS-induced impact ionization is dependent on the generation of electron-hole pairs, which is closely proportional to the channel layer thickness, test temperature, applied electric field, and drain current density. In other words, the structural design of the devices and the control of test conditions should be well considered to promote the stability performance of the devices.

## 4. Conclusions

In summary, the ITZO TFTs with various *T*_ITZO_ were fabricated and the DCS-induced instabilities under different test temperatures and *I*_DS_ values were investigated. All the TFT devices possessed excellent electrical properties irrespective of the *T*_ITZO_, including a high μ_sat_ of >35 cm^2^V^−1^s^−1^ and steep SS. For 100 nm *T*_ITZO_ devices evaluated at high *V*_GS_ and *V*_DS_ values, the transfer and output characteristics exhibited an abnormal rise in current. This phenomenon can be ascribed to the floating body effect that impacts ionization occurring near the drain region. More importantly, with the variation in the *T*_ITZO_, the synergistic effects of temperature and *I*_DS_ value on DCS-induced instability were well understood. The results further demonstrate that the abnormal negative shift behavior in TFT devices with the *T*_ITZO_ of greater than 75 nm is gradually enlarged with the increase in temperature and *I*_DS_ values. This study provides a method with which to comprehend the DCS-originating degradations for high-performance ITZO-based TFT devices.

## Figures and Tables

**Table 1:** The electrical parameters of a-ITZO TFTs with various *T*_ITZO_ extracted from the forward sweep.

Thickness (nm)	45	75	100
*μ*_lin_ (cm^2^∙V^−^^1^∙s^−^^1^)	28.76	33.27	46.36
*μ*_sat_ (cm^2^∙V^−^^1^∙s^−^^1^)	35.23	46.90	130.10
*V*_ON_ at *I*_DS_ = 1 nA (V)	1.19	0.43	−0.33
Hysteresis Δ*V*_H_ (V)	0.22	0.07	−6.38
Subthreshold swing (mV/dec.)	169	130	88
